# Gender disparity in cardiovascular mortality following radiation therapy for Hodgkin’s lymphoma: a systematic review

**DOI:** 10.1186/s40959-020-00067-7

**Published:** 2020-08-05

**Authors:** Yaser Khalid, Michael Fradley, Neethi Dasu, Kirti Dasu, Ankit Shah, Adam Levine

**Affiliations:** 1 Department of Internal Medicine, Memorial Healthcare System, Hollywood, FL USA; 2 Cardio-Oncology Program, Division of Cardiovascular Medicine, Abramson Cancer Center, Perelman School of Medicine at the University of Pennsylvania, Philadelphia, USA; 3 Department of Gastroenterology, Jefferson Health System NJ, Stratford, NJ USA; 4 Division of Biology, Syracuse University, Syracuse, NY USA; 5 Division of Cardiovascular Medicine, Rowan University School of Osteopathic Medicine at Jefferson Health System, Stratford, NJ USA; 6 Division of Interventional Cardiology, Virtua Lourdes Health System, Camden, NJ USA

**Keywords:** Radiation, Hodgkin’s lymphoma, Coronary artery disease, Women, Prevention

## Abstract

**Background:**

Radiation-induced coronary artery disease (R-CAD) has become an increasingly recognized phenomenon. Although the clinical relationship between radiation therapy and CAD risk is well known, there is minimal investigation of the gender relationship to radiation-induced CAD events and the resulting cardiovascular (CV) events/mortality. We study the gender variation in the incidence of CV events/mortality related to R-CAD in Hodgkin’s Lymphoma (HL) patients.

**Methods:**

The Preferred Reporting Items for Systematic Reviews and Meta-Analyses (PRISMA) guidelines were used in this systematic review and network meta-analysis. OVID, Cochrane Central Register of Controlled Trials via the Wiley Interface, Web of Science Core Collection, MEDLINE, EMBASE, and Google Scholar were investigated to identify prospective and retrospective observational studies comparing women and men following radiation treatment for Hodgkin’s lymphoma. Ten studies were included (4 prospective, 6 retrospective). The primary outcome was incidence of cardiovascular events/mortality. The secondary outcome was all-cause mortality. Meta-regression for age was also performed.

**Results:**

Of 13,975 patients, including 41% females and 59% males, CV events/mortality were noted to be significantly higher in women compared to men (OR 3.74, 95% CI 2.44–5.72, *p* < 0.001). All-cause mortality was also higher in women compared to men (OR 1.94, 95% CI 1.10–3.44, *p* < 0.023). On meta-regression analysis, elderly populations have a higher rate of mortality, which was even higher for women than men (coefficient = 0.0458, *p* = 0.0374).

**Conclusions:**

Women have a higher rate of R-CAD related CV events/mortality and all-cause mortality compared to men amongst radiation-treated patients. These data highlight the need for increased surveillance to better monitor for R-CAD in female patients treated with mantle or mediastinal radiation.

## Introduction

Radiation-induced coronary artery disease (R-CAD) has become an increasingly well recognized entity. R-CAD is one of the leading causes of morbidity and mortality amongst patients treated with radiotherapy for mediastinal malignancies, especially breast cancer and Hodgkin’s lymphoma (HL) [1, 2]. The risk of R-CAD increases with the frequency and duration of radiation therapy and the amount of radiation used [1–5].

Of note, approximately 8480 people were diagnosed with HL in 2020 in the United States [3–5]. Nearly half of the patients do not have complete response to chemotherapy alone and require further salvage treatment with radiotherapy and/or autologous stem cell transplant for treatment [6, 7]. For decades, mantle or mediastinal field radiotherapy has been a therapeutic option with cure rates shown to be as high as 85%, however, this therapy inadvertently leads to potential exposure of cardiac structures to radiation [3, 5]. Patients who have survived such radiation treatments are now presenting with higher rates of R-CAD related events, such as myocardial infarction, heart failure, ischemic cardiomyopathy, and/or arrythmias, with complications generally occurring 5–10 years or longer after treatment [3–6, 8–10].

There are minimal studies examining the role of gender, especially as an independent, non-traditional risk factor for increased cardiovascular (CV) events/mortality among patients treated with radiation. In this systematic review and meta-analysis, we assess the role of gender in the incidence of CV events/mortality related to R-CAD in Hodgkin’s Lymphoma (R-HL) patients who were treated with radiation.

## Methods

### Search methods and study selection

A systematic review and meta-analysis were performed following the Preferred Reporting Items for Systematic Reviews and Meta-Analyses (PRISMA) guidelines. Two co-authors (YSK and NRD) independently searched published studies indexed in OVID, Cochrane Central Register of Controlled Trials via the Wiley Interface, Web of Science Core Collection, MEDLINE, EMBASE, and Google Scholar from October 31, 2019 until April 1, 2020 thoroughly for the following keywords: “Radiation-Induced Coronary Artery Disease, Hodgkin’s Lymphoma, Radiation, Mediastinal Tumors.” Reviews, titles, abstracts, editorials were closely evaluated for the unrelated articles, which were subsequently excluded from our study. Research studies and articles published in all languages were considered.

All retrospective and prospective studies examining the rate of R-CAD in patients with HL were considered eligible. Only studies studying R-CAD with gender statistics were included. Adverse events, such as cardiovascular mortality and events, were defined by the standards of the Common Terminology Criteria for Adverse Events. The definition of cardiovascular events was a cardiac disorder that limited activities of daily living (ADLs) or proved to be life-threatening.

### Data extraction

Furthermore, two co-authors (YSK and NRD) independently screened studies for the inclusion. In Excel, a data extraction table was utilized to compile and display the pertinent information from each article, which included the following: first author’s last name, publication year, study design, country, duration of radiation treatment, sample size, number of patients for each gender, mean age of patients, median radiation dose, mortality from cardiovascular diseases or all-causes, and incidence of cardiovascular events (including myocardial infarction, abnormal stress test, received revascularization via coronary intervention or bypass, stroke, ventricular arrhythmias, heart failure, pericardial or valvular heart disease, and/or the presence of carotid intimal media thickness) were recorded. The table was constructed by the first author (YSK) and verified by one of the co-authors (NRD).

Finally, the Cochrane Risk of Bias Tool was employed to assess the risk of bias. Disagreements throughout this process were resolved by consensus of the entire team. When needed data was not directly found in the published articles, we obtained such data from the authors through response letters/e-mail or via reviewing their supplemental reports. All of the authors were contacted.

### Meta-analysis

The Hartung-Knapp-Sidik-Jonkman (HKSJ) method [11–13] was employed to complete the statistical analysis using MedCalc software (MedCalc Software Ltd., Belgium) with: 1) a summary of data from individual studies; 2) an investigation of the studies' heterogeneity graphically and statistically; 3) calculation of clustered indexes; and 4) graphical illustration via Forest Plots. Our assumption of heterogeneity was tested for each planned analysis using the Cochrane-Q heterogeneity and I^2^ statistics with each of the following values: low (0–25%), moderate (25–75%), and high (> 75%). Random effects models using the Mantel-Haenszel method and fixed effects models were both used [14, 15]. Meta-regression was analyzed using a generalization of Littenberg and Moses Linear model [16, 17] by inverse of the variance or study size unweighted. A secondary analysis was performed using the Der Simonian & Laird method and fixed effects models [18] through the Comprehensive Meta-Analysis software (Additional file 1: Appendix A).

## Results

### Baseline patient characteristics of included studies

**Fig. 1:**
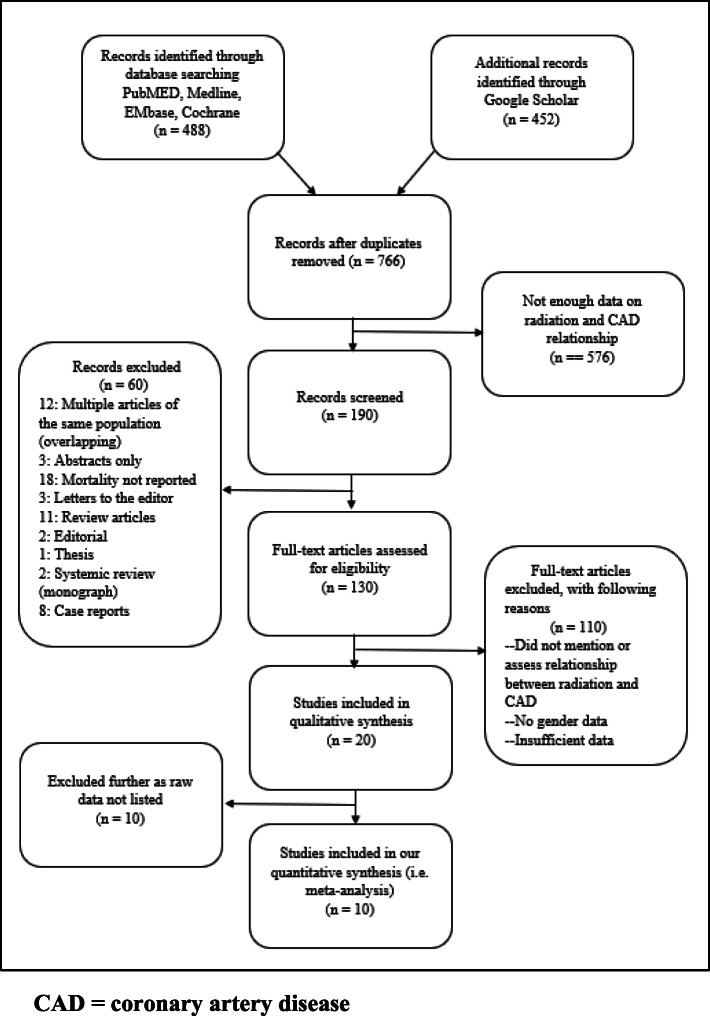
Prisma flow chart of studies screened and included in meta-analysis

Figure 1 displays the methods by which the studies were chosen for our analysis. At first, literature investigation yielded a total of 488 studies that were considered for inclusion in the study. Review articles, case reports, retrospective studies, abstracts, studies with insufficient data, and articles with overlapping study populations and redundant data were removed. Then, a total of 10 studies were evaluated in the final analysis (Table 1). All studies were conducted in the USA, Europe, and Canada. Four studies were retrospective studies and 6 were prospective studies. The number of patients in each study varied, with over 6000 patients in the largest study and 40 patients in the smallest study. Among a total of 13,975 patients (41% females and 59% males) from 10 studies with both patients with active cancer and cancer survivors, there was an average age of 40 years.

**Table 1:** List of Selected Studies with Their Demographics, Duration, Sample Size, Mean Age, Radiation Doses, and Cardiovascular Outcomes

Trial Information	Country	Duration	Sample (%)		Mean Age (Years)	Median Radiation Dose (Gy)	Outcome
			Female	Male			
**Boivin 1982, **[19] **Retrospective study**	USA	33.0 years	44.2	55.8	< 44	> 30	CV mortality
**Galper 2011, **[2] **Retrospective study**	USA	14.7 years	53.6	46.4	47	> 36	CAD (MI, CABG, PTCA), arrhythmias (AICD), valvular disease, pericardial disease
**Giancolo 2010, **[20] **Prospective study**	Italy	5.0 months	46.4	53.6	47.8 +/− 17.4	41.2+/−15.6	CIMT
**Hahn 2017, **[21] **Retrospective study**	Canada	One time analysis	39.2	60.8	34.2	25	Ischemic CVD
**Heidenreich 2007, **[22] **Prospective study**	USA	One time analysis	45.4	54.6	42	> 35	Asymptomatic CAD stress testing, followed by coronary angiography if positive stress test
**Hoppe 1997, **[23] **Prospective study**	USA	35.0 years	41.0	59.0	NA	> 30	Cardiac heart disease (pericarditis, coronary artery disease, functional valvular/conduction defects)
**Hull 2003, **[10] **Retrospective study**	USA	11.2 years	39.0	61.0	25	33	CAD (MI, CABG, PCI, > 75% stenosis)
**Schellong 2010, **[24] **Prospective study**	Germany, Austria	29.4 years	41.7	58.3	27.9	> 30	Cardiac heart disease (pericarditis, coronary artery disease, functional valvular/conduction defects)
**Swerdlow 2007, **[25] **Prospective study**	UK	25.0 years	38.1	61.9	34	> 30	CVD mortality
**Tsai 2011, **[26] **Prospective study**	Norway	20.0–24.0 years	77.5	22.5	51 +/− 9	41	LV wall motion and EF, and CAD

*Abbreviations: UK* United Kingdom, *CVD* Cardiovascular Disease, *CAD* Coronary Artery Disease, *MI* Myocardial Infarction, *CABG* Coronary Artery Bypass Graft, *PTCA* Percutaneous Coronary Balloon Angiogram, *AICD* Automatic Intrac-cardiac Defibrillator, *CIMT* Common Carotid Intima-Media Thickness, *PCI* Percutaneous Coronary Intervention, *LV* Left Ventricle, *EF* Ejection Fraction

### Rates of cardiovascular events and mortality

**Fig. 2:**
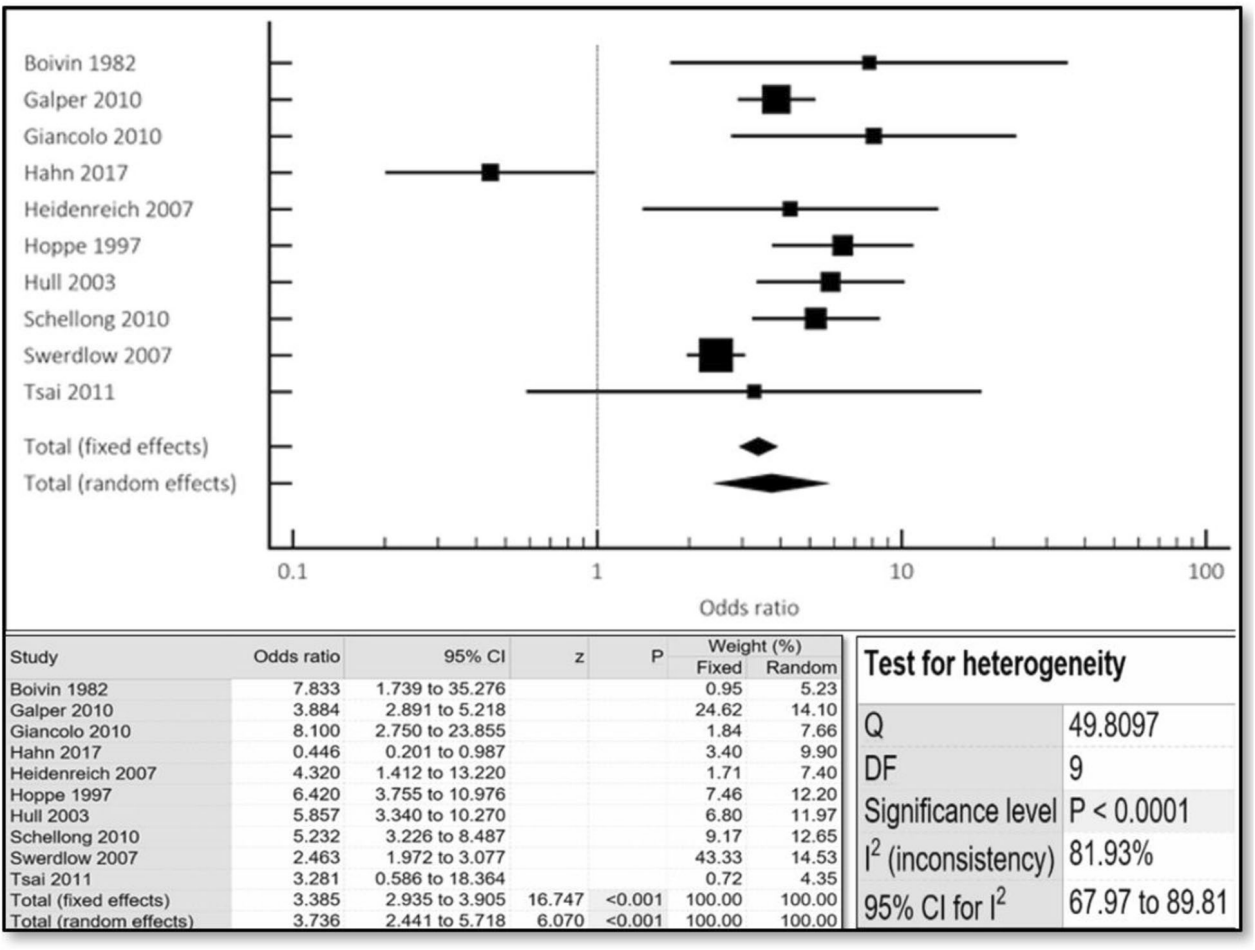
Cumulative incidence of cv events/mortality noted in r-hl females compared to males via combined fixed and random effects. Odds ratio, confidence interval and weight of studies for cv events/mortality for r-hl females compared to males via combined fixed and random effect. Test for heterogeneity of studies for r-hl females compared to males via combined fixed and random effects

**Fig. 3:**
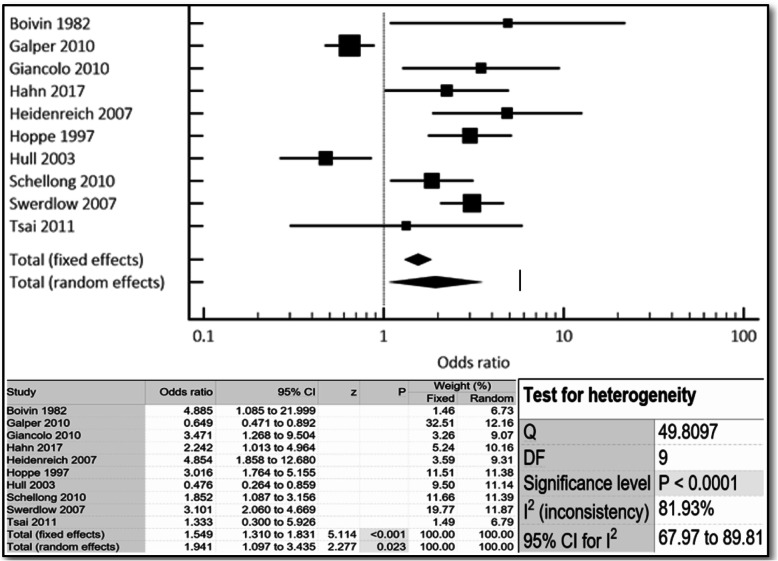
Cumulative incidence of all-cause mortality noted in r-hl females compared to males via combined fixed and random effects. Odds ratio, confidence interval and weight of studies for all-cause mortality for r-hl females compared to males via combined fixed and random effects. Test for heterogeneity of studies for r-hl females compared to males via combined fixed and random effects

The aggregate incidence of both cardiovascular events and mortality was approximately 4-times higher in women compared to men (OR 3.74, 95% CI 2.44–5.72, *p* < 0.001) (Fig. 2). All-cause mortality was also almost 2-times higher in women compared to men (OR 1.94, 95% CI 1.10–3.44, *p* < 0.023) (Fig. 3). Similar results were noted both in the fixed- and random-effects models.

**Fig. 4:**
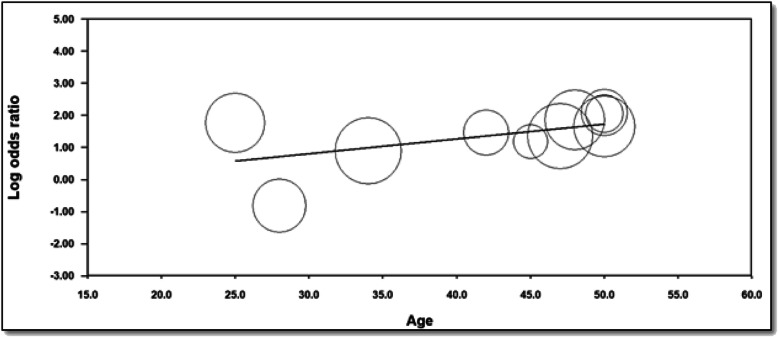
Meta-regression to assess for incidence of cv events/mortality of radiation treated female patients with aging

On meta-regression analysis (Fig. 4), both groups had higher mortality with advancing age, but this was even higher for women with definite increase of CV events/mortality at approximately 50 years of age that was statistically significant (coefficient = 0.0458, *p* = 0.0374).

Funnel plot analysis (Additional file 1: Appendix C) displayed no asymmetry in the assessed outcomes (*p* < 0.05 by Begg and Mazumdar’s test or Egger’s test). We have also performed Q and I^2^ analyses to assess for heterogeneity (Additional file 1: Appendix B).

## Discussion

Currently, there is a paucity of studies specifically addressing gender disparity in CV mortality after radiation treatment for HL. Our study is the first systematic review and meta-analysis evaluating this disparity. Previously, mantle field or mediastinal radiation has been proven in several studies to significantly raise the risk for cardiovascular diseases independently of traditional cardiac risk factors as well [4, 8]. Our analysis revealed that for women, such radiation therapy significantly raises CV events and mortality by almost four-fold and raises all-cause mortality by almost 2-fold. This could be due to the underrepresentation of women in these clinical trials, likely higher doses of radiation needed to treat the Hodgkin’s lymphoma for women, and a high degree of microvascular CAD in women.

Interestingly, studies have shown that the risk of CAD and related CV mortality increases when there are both traditional CV risk factors and radiation exposure [8, 10]. For example, diabetes has been linked increased hospitalizations and hypertension and hyperlipidemia were two times as likely to develop ischemic cardiac disease among patients with R-HL [8, 22]. Our study further demonstrated that with aging, the incidence of CV events markedly increased for female patients. All the studies evaluated in this meta-analysis were independently corrected for the traditional cardiac risk factors for CAD, like diabetes, hyperlipidemia, hypertension, and tobacco use. However, most of the studies did not clearly comment on the use of specific medical therapies for these conditions.

Additionally, high-dose mediastinal irradiation (cumulative dose of 35–40 Gy), increases cardiovascular disease and mortality in long-term survivors [24, 26, 27]. Mediastinal radiation treatment incidentally exposes a large quantity of the heart to radiation within the field, leading to increased risk of endothelial damage and promotion of atherosclerosis [25, 28]. In our analysis, most patients received over 30 Gy of radiation (Table 1). It should be recognized that in recent years, alterations to procedures and reductions in the radiation field volume have led to a significant reduction in cardiac exposure resulting in less cardiotoxicity than previously identified [3, 21].

The National Comprehensive Cancer Network currently recommends the following cardiovascular screening guidelines for survivors of HL to proactively reduce cardiovascular risk: annual blood pressure measurements, lipid panels, and serial serum glucose levels [23, 29, 30]. The American College of Radiology Appropriateness Criteria Expert Panel on Hodgkin Lymphoma Follow-up has broad recommendations: a stress test and an echocardiogram every 5 to 10 years after treatment as appropriate [1]. We suggest the development of specific screening guidelines for high risk patient groups treated with mantle field or mediastinal radiation, including female gender, radiation doses over 30 Gy, concomitant use of cardiotoxic chemotherapy, and/or having one or more traditional CV risk factors.

### Study limitations

We acknowledge certain limitations associated with this study. Since this is a study-level analysis, it is not possible to make definitive conclusions about gender risks for patients with R-CAD. We identified only 10 retrospective and prospective observational cohort studies, accounting for an overall small sample size. None of the selected studies matched men and women with HL to radiation therapy dose, age of HL diagnosis, and how CVD was diagnosed. The studies did not specify the frequency and duration of radiation therapy for their patient populations. These findings may have led to a different course of CAD and subsequently CV mortality. Our study population mostly consisted of Caucasian nations. Most of the selected studies included young patients with a median age of 40 years. Unfortunately, most of the studies did not list when radiation was administered, total radiation dose received (only the average radiation dose), side of chest irradiated, concomitant chemotherapy, follow-up years after radiation therapy, methods to shield the heart during radiation, and presence of CV risk factors. All of these factors could affect the premature occurrence of radiation-induced CAD.

## Conclusions

The risk of cardiovascular events and mortality is substantial for women with HL and R-CAD. Moreover, the rate of all-cause mortality was also higher in women compared to men. The findings demonstrate the need for vigilant screening for cardiovascular disease among cancer patients and survivors who have received mediastinal radiation. Future studies and analyses must focus on age, dose, duration of treatment, the mode of diagnosis for CVD as well as matching men and women with HL to radiation therapy dose, age at diagnosis, method of diagnosing CVD, and specify dose, frequency, and duration of radiation therapy. This will would allow researchers to make definitive conclusions regarding gender risks.

## Supplementary information

**Additional file 1.**

## Data Availability

Available.

## Abbreviations

R-CAD: radiation-induced coronary artery disease
HL: Hodgkin’s lymphoma
CV: cardiovascular
OR: odds ratio
CI: confidence interval
