# Supplementary material for: Gender disparity in cardiovascular mortality following radiation therapy for Hodgkin’s lymphoma: a systematic review
Source: Cardiooncology. 2020 Aug 5;6:12. doi: 10.1186/s40959-020-00067-7 (PMC7405444; doi:10.1186/s40959-020-00067-7)

**APPENDIX A**

**
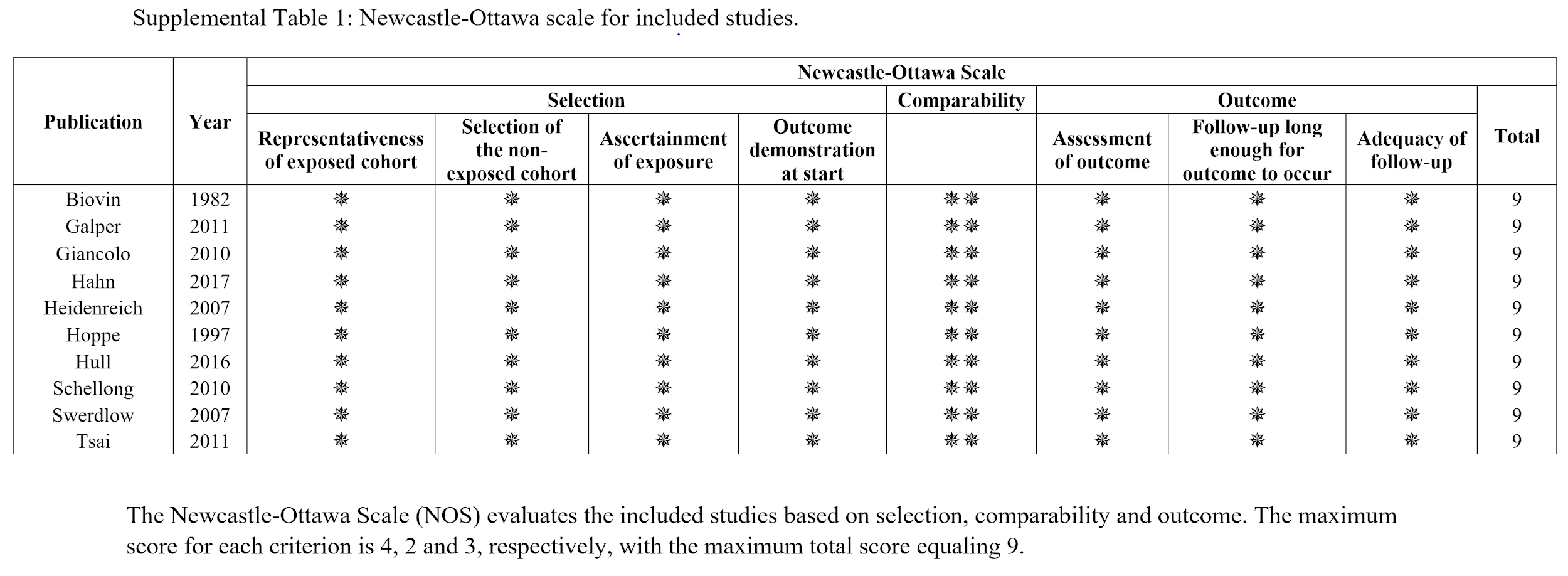
**


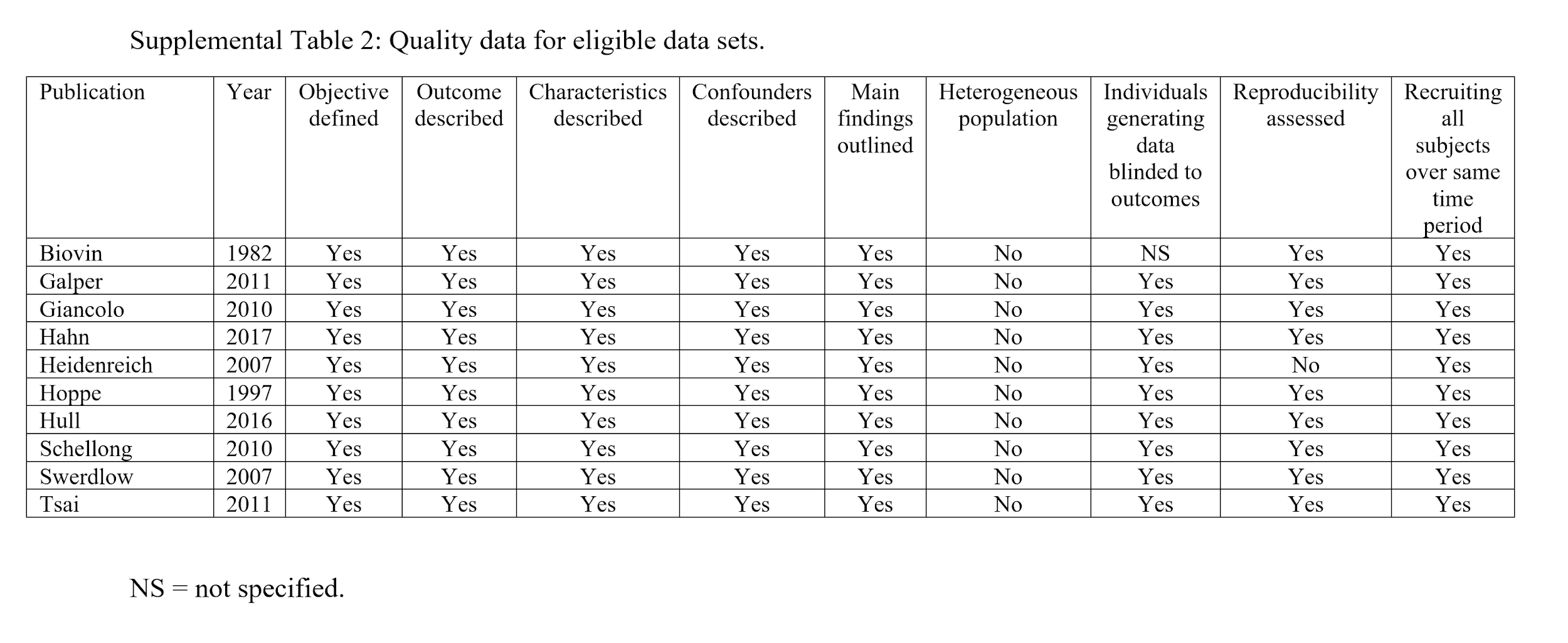


**APPENDIX B**

**
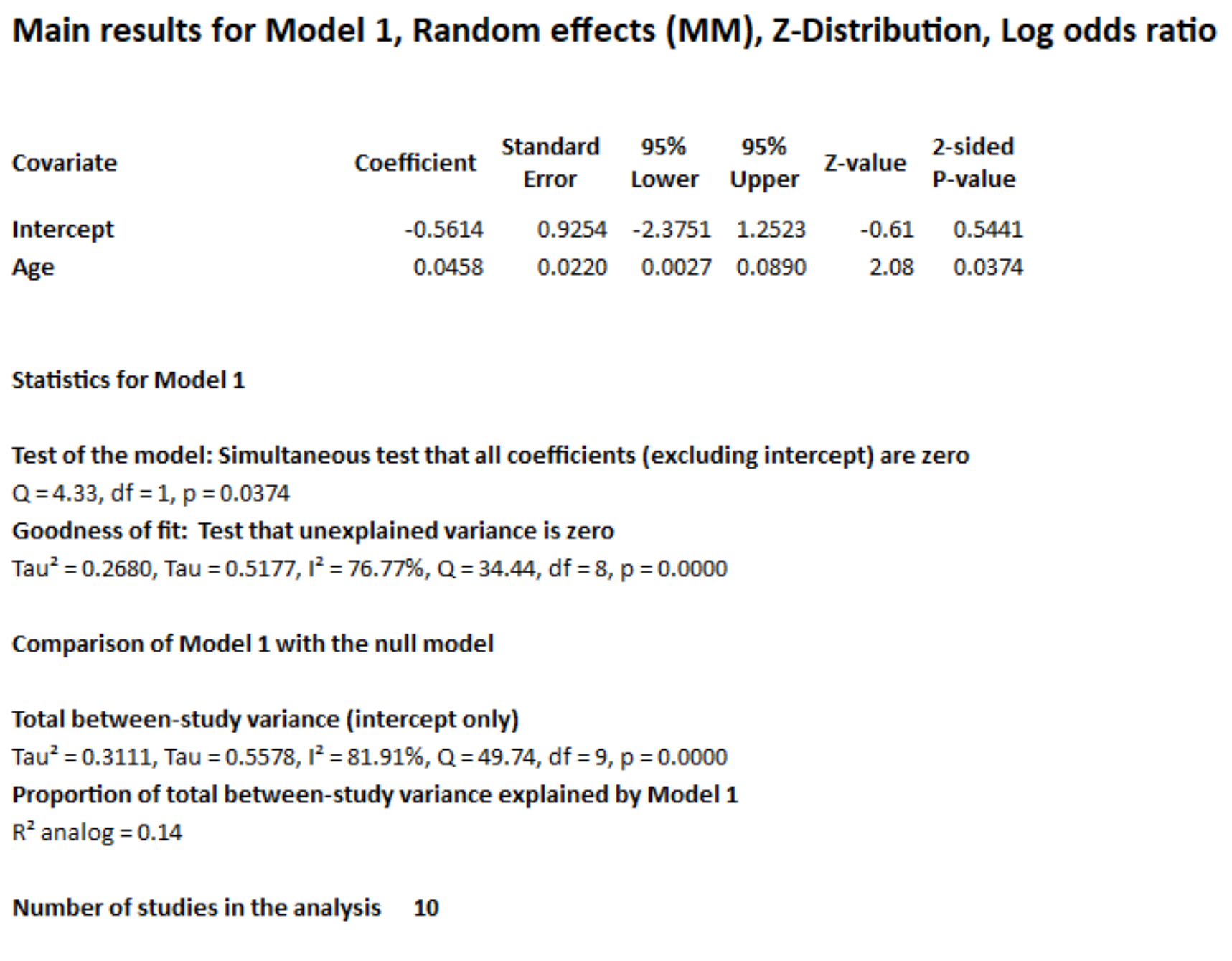
**

**Appendix C:**

**FUNNEL PLOT ANALYSIS TO ASSESS FOR POTENTIAL PUBLICATION BIAS AND/OR PRESENCE OF HETEROGENEITY FOR CVD**


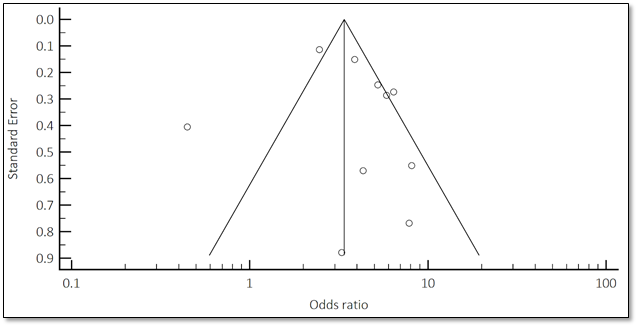


**FUNNEL PLOT ANALYSIS TO ASSESS FOR POTENTIAL PUBLICATION BIAS AND/OR PRESENCE OF HETEROGENEITY FOR ALL-CAUSE MORTALITY**


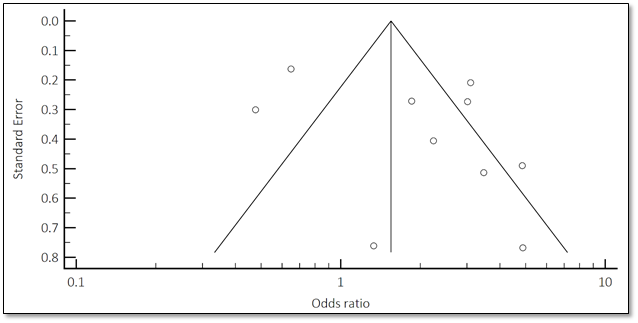

Supplement: Supplementary file 1 — Additional file 1. [file 40959_2020_67_MOESM1_ESM.docx]
